# Nighttime is associated with decreased survival and resuscitation efforts for out-of-hospital cardiac arrests: a prospective observational study

**DOI:** 10.1186/s13054-016-1323-4

**Published:** 2016-05-10

**Authors:** Yosuke Matsumura, Taka-aki Nakada, Koichiro Shinozaki, Takashi Tagami, Tomohisa Nomura, Yoshio Tahara, Atsushi Sakurai, Naohiro Yonemoto, Ken Nagao, Arino Yaguchi, Naoto Morimura, SOS-KANTO 2012 study group

**Affiliations:** Department of Emergency and Critical Care Medicine, Chiba University Graduate School of Medicine, 1-8-1 Inohana, Chuo-ku, Chiba City, Chiba 260-8677 Japan; Department of Emergency and Critical Care Medicine, Nippon Medical School Hospital, 1-1-5 Sendagi Bunkyo-ku, Tokyo, 113-0022 Japan; Department of Emergency and Critical Care Medicine, Juntendo University Nerima Hospital, 3-1-10 Takanodai, Nerima-ku, Tokyo 177-0033 Japan; National Cerebral and Cardiovascular Center Hospital, 5-7-1 Fujishiro-dai, Suita, Osaka 565-8565 Japan; Division of Emergency and Critical Care Medicine, Department of Acute Medicine, Nihon University School of Medicine, 30-1 Oyaguchikamicho, Itabashi-ku, Tokyo 173-0032 Japan; Department of Biostatistics, Kyoto University School of Public Health, Kyoto, 606-8501 Japan; Nihon University Surugadai Hospital, 1-6 Kanda-Surugadai, Chiyoda-ku, Tokyo 101-8309 Japan; Department of Critical Care and Emergency Medicine, Tokyo Women’s Medical University, 8-1 Kawadacho, Shinjuku-ku, Tokyo 162-8666 Japan; Department of Emergency Medicine, Yokohama City University Medical Center, 4 -57 Urafunecho, Minami-ku, Yokohama City, Kanagawa 232-0024 Japan

**Keywords:** Cardiopulmonary resuscitation, Circadian rhythm, Heart arrest, Resuscitation, Out-of-hospital cardiac arrest

## Abstract

**Background:**

Whether temporal differences alter the clinical outcomes of patients with out-of-hospital cardiac arrest (OHCA) remains inconclusive. Furthermore, the relationship between time of day and resuscitation efforts is unknown.

**Methods:**

We studied adult OHCA patients in the Survey of Survivors after Out-of-Hospital Cardiac Arrest in the Kanto Region (SOS-KANTO) 2012 study from January 2012 to March 2013 in Japan. The primary variable was 1-month survival. The secondary outcome variables were prehospital and in-hospital resuscitation efforts by bystanders, emergency medical services personnel, and in-hospital healthcare providers. Daytime was defined as 0701 to 1500 h, evening was defined as 1501 to 2300 h, and night was defined as 2301 to 0700 h.

**Results:**

During the study period, 13,780 patients were included in the analysis. The patients with night OHCA had significantly lower 1-month survival compared to the patients with daytime OHCA (night vs. daytime, adjusted odds ratio (OR) 1.66; 95 % confidence interval (CI), 1.34–2.07; *P* < 0.0001). The nighttime OHCA patients had significantly shorter call-response intervals, bystander CPR, in-hospital intubation, and in-hospital blood gas analyses compared to the daytime and evening OHCA patients (call-response interval: OR 0.95 and 95 % CI 0.93–0.96; bystander CPR: OR 0.85 and 95 % CI 0.78–0.93; in-hospital intubation: OR 0.85 and 95 % CI 0.74–0.97; and in-hospital blood gas analysis: OR 0.86 and 95 % CI 0.75–0.98).

**Conclusions:**

There was a significant temporal difference in 1-month survival after OHCA. The nighttime OHCA patients had significantly decreased resuscitation efforts by bystanders and in-hospital healthcare providers compared to those with evening and daytime OHCA.

**Electronic supplementary material:**

The online version of this article (doi:10.1186/s13054-016-1323-4) contains supplementary material, which is available to authorized users.

## Background

Out-of-hospital cardiac arrest (OHCA) is a major public health problem worldwide [1, 2]. Despite the recent advances in the management of OHCA, the fatality rate remains unsatisfactory [3, 4]. The Utstein-style guidelines have shown that key elements alter clinical outcomes of patients with OHCA, such as system factors, dispatch/recognition, patient variables, and resuscitation/postresuscitation processes [5, 6], and these vary according to time of day. Shift work disrupts circadian rhythms, causing health risks and deterioration in the worker’s ability and performance in terms of attention, motivation, and decision-making [7–9]. Thus, nighttime circumstances may influence the resuscitation efforts performed by healthcare providers [10, 11] when OHCA occurs.

To our knowledge, five investigations of time differences and clinical outcomes of patients with OHCA did not yield consistent conclusions [12–16]. The two reports showed no significant differences [12, 16]. Brooks et al. reported no significant temporal difference in survival to hospital discharge in the US/Canadian population (time period, adjusted odds ratio (OR)(95 % confidence interval (CI)) compared to 0001–0600 h; 0601–12:00 h, 0.99 (0.74–1.34); 1201–1800 h, 1.08 (0.84–1.54); 1801–2400 h, 0.96 (0.71–1.29) [12]. In an Austrian study Uray et al. observed no significant difference in 12-month survival between daytime (0800–1600 h) and nighttime (1601–0759 h) admission (daytime, 48.6 % vs. nighttime, 48.3 %; *P* = 0.94) [16]. This is the latest among the five studies published but it is based on a single specialized resuscitation center. However, three large-scale studies performed in Japan and the USA showed that the patients who had OHCA during the night had increased mortality compared to those with daytime OHCA [13–15]. Koike et al. observed lower 1-month survival in the nighttime (1700–0859 h) (OR (95 % CI) 1.26 (1.22–1.31)) [13]. Wallace et al. reported lower 30-day survival in nighttime (2000–0759 h) (OR (95 % CI) 1.10 (1.02–1.18)) [14]. Bagai et al. observed deceased hospital discharge in overnight (2301–0700 h) compared to daytime (0:01–15:00 h) (OR (95 % CI) 1.23 (1.06–1.43)) [15]. Furthermore, the associations between time of day and the  resuscitation efforts remain insufficiently tested.

Because resuscitation is associated with altered clinical outcomes after cardiac arrest (CA) [10], the evaluation of temporal differences in prehospital or in-hospital resuscitation efforts is of great interest to improve patient outcomes after OHCA.

Thus, we first tested the hypothesis that patients who had OHCA during the nighttime had worse clinical outcomes when compared to patients who had OHCA during day or evening hours. We further tested for the association between nighttime OHCA with differences in the resuscitation efforts by bystanders and healthcare providers, using a large, multicenter cohort. The primary outcome variable was 1-month survival. The secondary outcome variables included prehospital and in-hospital resuscitation efforts.

## Methods

### Study design

The Survey of Survivors after Out-of-Hospital Cardiac Arrest in the Kanto Region (SOS-KANTO) 2012 study was prospectively conducted to accumulate prehospital and in-hospital records of patients who had CA, by emergency medical services (EMS) personnel and hospital staff in the Kanto region, including Tokyo Prefecture, Japan, with the support of the Kanto Regional Group of the Japanese Association for Acute Medicine [17]. The SOS-KANTO 2012 study included 16,452 CA patients from 67 emergency medical centers between January 2012 and March 2013. The relevant institutional review boards of all institutions approved the study (see Additional file 1 for more detail). The review boards waived the need for written informed consent.

### Study populations

**Fig. 1:**
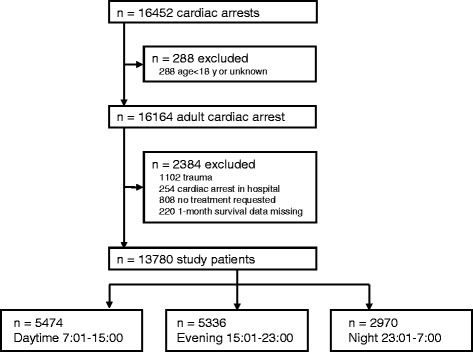
Flow diagram of the study population. A total of 16,452 cardiac arrest patients were enrolled in the Survey of Survivors after Out-of-Hospital Cardiac Arrest in the Kanto Region (SOS-KANTO) 2012 study. Of the initial cohort of 16,452 patients with out-of-hospital cardiac arrest (OHCA), 288 were excluded because they were <18 years old, leaving 16,164 adult patients. Of these, 2,384 were excluded, resulting in a study cohort of 13,780 patients included in the analysis. Patients were assigned to three categories according to the time of the 911 (emergency) call receipt: daytime (0701–1500 h), evening (1501–230 h), and night (2301–0700 h)

The current study included patients aged 18 years and older who fit the following criteria: EMS-treated CA and subsequently transported to one of the participating institutions. Exclusion criteria were as follows: (1) CA caused by trauma; (2) CA that occurred in hospital; (3) advanced life support declined by family members when the patient arrived at the hospital; and (4) unknown main outcomes (1-month survival) (Fig. 1).

### Data collection and definition

EMS personnel collected prehospital information in the standardized Utstein-style template, including age, sex, location, witnessed CA, bystander cardiopulmonary resuscitation (CPR), call-response interval, initial cardiac rhythms monitored by EMS personnel, prehospital advanced airway, adrenaline administration, defibrillation, and prehospital return of spontaneous circulation (ROSC). The call-response interval was defined as the interval from the 911 (emergency) call receipt by emergency response dispatchers to the time when the emergency response vehicle arrived on scene. Patients were assigned to three categories according to the time of the receipt of a 911 call regardless of the witness status: daytime (0701–1500 h), evening (1501–2300 h), and night (2301–0700 h) [15]. In Japan, EMS personnel work in 24-h shifts and are obligated to provide patients with nationally uniform protocols. They can place laryngeal tubes or laryngeal mask airways. Only certified EMS personnel can perform endotracheal intubation and the number of such personnel is still limited.

Physicians who were responsible for treatments determined the causes of CA. The institutional researchers collected information that included cardiac rhythms/ROSC on hospital arrival, in-hospital intubation, adrenaline administration, defibrillation, blood gas analysis, ROSC during resuscitation by physicians (in-hospital ROSC), 24-h survival, 1-month survival, and neurological outcomes. Data registered by the institutional researchers were independently checked by the data and safety monitoring committee members in the SOS-KANTO 2012 study group, who created the dataset for analysis.

### Statistical analysis

We tested for differences in baseline characteristics and clinical outcomes by time of day using the chi-squared test for categorical data and the Kruskal-Wallis/Mann-Whitney *U* test for continuous data. For the primary analysis of 1-month survival, we chose a multivariate analysis to test for temporal differences (daytime vs. evening vs. night) in 1-month survival to allow for adjustment of potential confounding factors based on a previous study [15], including age, sex, witness status, bystander CPR, call-response interval, and initial shockable rhythm. We adjusted possible clustering effects of institutions using a generalized estimating equation. The secondary outcome variables were call-response interval, proportion of bystander CPR, intubation or advanced airway, adrenaline administration, defibrillation, and blood gas analysis provision. For the secondary analysis, we evaluated the nighttime effect (vs. daytime and evening) on prehospital and in-hospital resuscitation efforts. We chose multivariate logistic regression analysis to evaluate prehospital resuscitation efforts to allow for adjustment of confounding factors that are the same as in the primary analysis. On analysis of in-hospital resuscitation efforts, we further added adjustments for ROSC on hospital arrival as a covariate because patient with ROSC on arrival may have decreased chances of adrenaline administration and defibrillation. Possible clustering effects of institutions were adjusted using a generalized estimating equation. Adjusted OR and 95 % CI were calculated. The level of significance was set at α = 0.05 with a two-tailed test. Analyses were performed using IBM SPSS version 22 (IBM Corp., Armonk, NY, USA) statistical software packages.

## Results

**Fig. 2:**
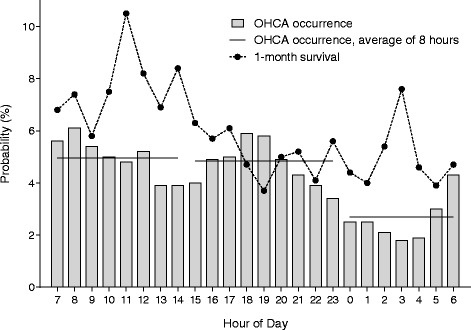
Occurrence of out-of-hospital cardiac arrest (*OHCA*) and 1-month survival after OHCA by hour of the day. There were temporal differences in OHCA occurrence with a bimodal distribution; OHCA less frequently occurred at night (2301–0700 h, 2.7 % hourly, average of 8 h) compared to daytime (0701–1500 h, 5.0 % hourly) and evening (1501–2300 h, 4.8 % hourly). Patients with daytime OHCA had a 1-month survival rate of 7.6 %, patients with evening OHCA had a 1-month survival rate of 5.0 %, and patients with nighttime OHCA had a 1-month survival rate of 4.9 %

Of the initial cohort of 16,452 OHCA patients, 288 were excluded because they were <18 years old, leaving 16,164 adult patients. Of these, 2,384 were excluded, resulting in a study cohort of 13,780 patients included in the analysis (Fig. 1). There were temporal differences in the incidence of OHCA; OHCA occurred less frequently during the nighttime (2301–0700 h) compared to during the daytime (0701–1500 h) and evening (1501–2300 h) (Table 1; Fig. 2). There were significant differences in baseline characteristics between the daytime, evening, and night groups (Table 1). Daytime OHCA patients had a greater 1-month survival rate (7.6 %) compared to evening (5.0 %) and nighttime OHCA patients (4.9 %) (Table 1; Fig. 2).

**Table 1:** Baseline characteristics and clinical outcome by time of day of cardiac arrest occurrence

	Time of 911 emergency call receipt			*P* value
	Daytime	Evening	Night	
	0701–1500 h	1501–2300 h	2301–0700 h	
	(n = 5474)	(n = 5336)	(n = 2970)	
Age, years	75 (63–83)	76 (64–84)	74 (61–83)	0.0003
Sex, % male	61.9	59.1	60.3	0.12
Location, % home	66.0	75.6	76.2	<0.0001
Witnessed arrest, %	51.6	47.4	46.4	<0.0001
Bystander CPR, %	37.3	37.8	33.6	0.0003
Call-response interval, minutes	7.0 (6.0–10)	7.0 (6.0–10)	7.0 (6.0–9.0)	<0.0001
Initial shockable rhythm, %	9.1	7.0	7.2	0.0001
Cardiac etiology, %	51.8	51.1	56.0	<0.0001
Prehospital ROSC, %	10.4	7.4	5.5	<0.0001
ROSC, %	38.2	32.3	29.6	<0.0001
24-h survival, %	15.3	10.8	9.9	<0.0001
1-month survival, %	7.6	5.0	4.9	<0.0001
1-month good recovery, %	4.7	2.9	3.1	<0.0001

Data are presented as the median (interquartile range) for continuous variables and absolute numbers (percentages) for categorical data. Call-response interval, the interval between call receipt and ambulance arrival on scene; initial shockable rhythm, ventricular fibrillation or pulseless ventricular tachycardia initially monitored by emergency medical services providers; 1-month good recovery, survival with favorable neurological outcome defined as cerebral performance category of 1 or 2 at 1 month after cardiac arrest. *P* values were calculated using the Kruskal-Wallis test and chi-squared test. *CPR* cardiopulmonary resuscitation, *ROSC* return of spontaneous circulation

We tested for the association between the time of the 911 call receipt and 1-month survival in a multivariate analysis using a generalized estimating equation as the primary analysis. Patients with nighttime OHCA had significantly lower 1-month survival compared to the patients with daytime OHCA (adjusted OR 1.66; 95 % CI 1.34–2.07; *P* < 0.0001) (Table 2).

**Table 2:** Multivariate analysis of 1-month survival using a generalized estimating equation

	Odds ratio (95 % confidence interval)	*P* value
Age per year	0.98 (0.97–0.98)	<0.0001
Male	0.83 (0.69–1.00)	0.52
Witnessed arrest	3.56 (2.83–4.46)	<0.0001
Bystander CPR	1.62 (1.33–1.98)	<0.0001
Call-response interval per minute	0.94 (0.92–0.96)	<0.0001
Initial shockable rhythm	5.33 (4.23–6.71)	<0.0001
Time of 911 call receipt		
Night (2301–0700 h)	Reference	
Daytime (0701–1500 h)	1.66 (1.34–2.07)	<0.0001
Evening (1501–2300 h)	1.12 (0.88–1.43)	0.36

Call-response interval, intervals between call receipt and ambulance arrival on-scene; initial rhythm shockable, ventricular fibrillation or pulseless ventricular tachycardia initially monitored by emergency medical service providers. *P* values were calculated using a generalized estimating equation in addition to the adjustment for the variables listed here. *CPR* cardiopulmonary resuscitation

We next tested whether nighttime OHCA was associated with differences in the resuscitation efforts. In the univariate analysis, patients with nighttime OHCA had lower implementation rates of bystander CPR, in-hospital intubation, and in-hospital blood gas analysis, and had greater implementation rates of in-hospital adrenaline administration compared to the patients with daytime and evening OHCA (Table 3). In the multivariate analysis to correct for potential confounding factors due to baseline imbalances, the patients with nighttime OHCA had significantly shorter call-response intervals, less bystander CPR, in-hospital intubation, and in-hospital blood gas analysis compared to the patients with daytime and evening OHCA (call-response interval: OR 0.95; 95 % CI 0.93–0.96; bystander CPR: OR 0.85; 95 % CI 0.78–0.93; in-hospital intubation: OR 0.85; 95 % CI 0.74–0.97; in-hospital blood gas analysis: OR 0.86; 95 % CI 0.75–0.98) (Table 4).

**Table 3:** Resuscitation efforts of patients with cardiac arrest occurring during daytime, evening and nighttime hours

	Time of 911 emergency call receipt		*P* value
	Daytime and evening	Night	
	0701–2300 h	2301–0700 h	
	(n = 10,810)	(n = 2970)	
Prehospital, *n* (%)			
Call-response interval	7 (6, 10)	7 (6, 9)	<0.0001
Bystander CPR	4044 (37.5)	994 (33.6)	0.0001
Advanced airway	4992 (48.6)	1414 (49.8)	0.280
Adrenaline	2153 (21.0)	539 (19.3)	0.055
Initial shockable rhythm	856 (7.9)	212 (7.1)	
Defibrillation	828 (96.7)	210 (99.1)	0.100
In-hospital, *n* (%)			
Intubation	8517 (86.8)	2299 (84.7)	0.006
Adrenaline	8883 (85.3)	2487 (87.5)	0.004
Shockable rhythm without ROSC	351 (3.2)	90 (3.0)	
Defibrillation	300 (85.5)	84 (93.3)	0.052
Blood gas analysis	9482 (89.6)	2539 (87.6)	0.002

We limited the analysis of prehospital defibrillation to the patients who had initially shockable rhythms during resuscitation by emergency services personnel, and the analysis of in-hospital defibrillation to patients who had shockable rhythm without return of spontaneous circulation (*ROSC*) on hospital arrival. Patients with prehospital intubation were excluded from the analysis of in-hospital intubation. Data are presented as the median (interquartile range) for continuous variables and absolute numbers (percentages) for categorical data. *P* values were calculated using the Mann-Whitney *U* test and chi-squared test. *CPR* cardiopulmonary resuscitation

**Table 4:** Association between resuscitation efforts and cardiac arrest occurring during nighttime hours

	Odds ratio (95 % confidence interval)	*P* value
Prehospital resuscitation		
Call-response interval	0.95 (0.93–0.96)	<0.0001
Bystander CPR	0.85 (0.78–0.93)	0.0002
Advanced airway	1.06 (0.97–1.15)	0.193
Adrenaline	0.92 (0.83–1.03)	0.152
Defibrillation	3.48 (0.81–14.9)	0.093
In-hospital resuscitation		
Intubation	0.85 (0.74–0.97)	0.019
Adrenaline	0.99 (0.85–1.16)	0.930
Defibrillation	2.14 (0.93–4.95)	0.074
Blood gas analysis	0.86 (0.75–0.98)	0.020

We limited the analysis of prehospital defibrillation to the patients who had initially shockable rhythm during resuscitation by emergency services personnel, and the analysis of in-hospital defibrillation to patients who had shockable rhythm without return of spontaneous circulation (*ROSC*) on hospital arrival. Patients with prehospital intubation were excluded from the analysis of in-hospital intubation. *P* values for prehospital resuscitation were calculated using multivariate logistic regression analysis corrected for age, sex, witness status, call-response interval, bystander cardiopulmonary resuscitation (*CPR*), and initial shockable rhythm. For the analysis of the in-hospital resuscitation, we further added ROSC on hospital arrival as a covariate and used a generalized estimating equation to account for possible clustering effects of institutions

## Discussion

We found that the patients who experienced OHCA during the nighttime had lower 1-month survival compared to those with daytime OHCA. The patients with nighttime OHCA had decreased resuscitation efforts by bystanders and in-hospital healthcare providers, including bystander CPR, in-hospital intubation, and in-hospital blood gas analysis compared to the daytime and evening OHCA patients.

The temporal variability by time of day was recognized by the internal circadian clock, which regulates a variety of biological processes, including cardiovascular physiology, according to an approximate 24-h period and is involved in the incidence of cardiovascular events [18], which can alter the etiology of OHCA or incidence of OHCA by time of day. In this study, OHCA less frequently occurred during the nighttime compared to daytime and evening; the incidence of OHCA had a bimodal distribution, which was consistent with previous work [12, 15, 19–21]. We also found that there were temporal differences in key elements listed in the Utstein-style guidelines [5, 6], such as age, location of arrest, witness status, bystander CPR, and initial rhythms.

In the present study, patients with nighttime OHCA had significantly lower survival compared to the patients with daytime and evening OHCA (OR 1.66; 95 % CI 1.34–2.07), which is consistent with three recent large-scale studies. Koike et al. defined 0900–1659 h as daytime and 1700 to 0859 h as nighttime, and observed lower 1-month survival for nighttime CA (OR 1.26; 95 % CI 1.22–1.31) [13]. Wallace et al. defined 0800 to 1959 h as daytime and 2000 to 0759 as nighttime, and reported lower 30-day survival for nighttime CA (OR 1.10; 95 % CI 1.02–1.18) [14]. Bagai et al. defined 0701–1500 h as daytime, 1501–2300 h as evening, and 2301–0700 h as overnight, and observed lower hospital discharge rates for overnight CA compared to daytime CA (OR 1.23; 95 % CI 1.06–1.43) [15]. Thus, temporal differences appear to alter OHCA outcome. However, Brooks et al. [12] and Uray et al. [16] reported no significant temporal difference in survival after OHCA after multivariate adjustment. Smaller patient sample sizes for nighttime OHCA (*n* = 569 and 1428) in these two studies compared to the other three studies (*n* = 1962, 4202, and 104,076) may yield inconsistent results.

During the prehospital period, bystander and EMS personnel contributed to the resuscitation efforts, which were subsequently taken over by in-hospital healthcare providers. We found that patients with nighttime OHCA received less bystander CPR and had shorter call-response intervals in prehospital settings. Our finding of less bystander CPR during nighttime hours was consistent with Wallace et al., who reported that bystander CPR was more likely to occur during the day, even after multivariate adjustment (adjusted relative risk 1.20; 95 % CI 1.13–1.28) [14]. Thus, there appears to be less bystander CPR performed in the community during the nighttime. We observed shorter call-response intervals in the nighttime although the median values for both time groups were the same (7 minutes). Wallace et al. reported longer call-response intervals during the night (median values for both time groups were the same at 6 minutes) [14]. The call-response interval could be partly involved in the EMS resuscitation, but it may be affected by other factors such as traffic conditions. As these two studies reported statistically opposite findings but with median values that were the same in both daytime and nighttime hours, these differences might be clinically subtle. Other than the call-response interval, we found no temporal differences between the nighttime and the daytime and evening hours, in prehospital resuscitation efforts by EMS personnel. Thus, the EMS personnel working in 24-h shifts consistently appear to provide prehospital care regardless of the time of day, according to the nationally uniform protocols.

Resuscitation efforts by in-hospital healthcare providers involved less intubation and blood gas analysis during the nighttime, which is in contrast to the consistent resuscitation provided by EMS personnel. This might be because of differences in work shifts between in-hospital care providers and EMS personnel; in-hospital care providers rarely work 24-h shifts in Japan [22]. The quantity of in-hospital human resources decreased during the nighttime, which may have caused operational differences [11]. These factors might contribute to the reduction in in-hospital resuscitation efforts during the nighttime. As adrenaline and defibrillation are recommended in advanced cardiovascular life support algorithm guidelines [23], these two might not be affected by operational differences among the four in-hospital resuscitation efforts in the current study. Lower rates of survival to hospital discharge for patients with in-hospital CA in the nighttime have also been reported [24, 25]. Because decreased resuscitation efforts could lead to poorer survival of patients with in-hospital CA, such as, for example, the duration of resuscitation efforts [10], less effective resuscitation by bystander and in-hospital healthcare providers might increase mortality among patients with nighttime OHCA. On the other hand, too many measures might be taken in the in-hospital process in the sense that intubation, adrenaline administration, defibrillation, and blood gas analysis were still performed in the vast majority of patients. Thus, statistically different in-hospital processes measures might not have significant relevance with clinical impact.

### Study limitations

This study has several limitations. First, our study cohort was not population-based, but was hospital-driven. However, the occurrence OHCA in this study had a bimodal distribution. This distribution was consistent with those of previously reported population-based studies [15, 21], suggesting universality of our cohort population. Second, our results showed significant temporal differences in survival after OHCA and less treatment during the nighttime. However, there was no causal link shown between lower survival and less treatment during the nighttime. Third, we used the emergency call receipt time as a surrogate for the time of cardiac arrest, therefore, it could be different from the actual collapse of the patient, particularly for unwitnessed OHCA that happened at nighttime [12–15, 26–28]. Fourth, all participating hospitals in this study are emergency medical centers to care for patients in the nighttime; however, we have no detailed data available on any operational differences between daytime and nighttime. Thus, we could not discuss the characteristics of operational differences between daytime and nighttime in the participating hospitals or compare the participating hospitals to other hospitals in Japan or other countries.

## Conclusions

In our large, observational study of temporal variability by time of day, there was a significant temporal difference in 1-month survival after OHCA. The patients who experienced OHCA during the nighttime (2301–0700 h) had significantly lower 1-month survival rates compared to those who experienced OHCA during the daytime. The patients with OHCA treated during the nighttime had lower implementation rates of bystander CPR, in-hospital intubation, and blood gas analysis compared to those with daytime and evening OHCA. Therefore, more intensive resuscitation efforts during the nighttime may improve patient survival after OHCA.

## Key messages

- The patients who experienced OHCA during the nighttime (2301–0700 h) had significantly lower 1-month survival rates compared to patients with OHCA during the daytime.
- As the patients with nighttime OHCA had a lower implementation rate of bystander CPR, in-hospital intubation, and blood gas analysis, increasing the resuscitation efforts during the nighttime may improve patient survival after OHCA.

## Additional file

Additional file 1: Table E1. The List of institutional reviewer boards approved the SOS-KANTO 2012 study. Online data supplement. (DOC 42 kb)

## Abbreviations

CA: cardiac arrest
CI: confidence intervals
CPR: cardiopulmonary resuscitation
EMS: emergency medical services
OHCA: out-of-hospital cardiac arrest
ROSC: return of spontaneous circulation, OR, odds ratios
SOS-KANTO: Survey of Survivors after Out-of-Hospital Cardiac Arrest in the Kanto Region
