# Supplementary material for: Nighttime is associated with decreased survival and resuscitation efforts for out-of-hospital cardiac arrests: a prospective observational study
Source: Crit Care. 2016 May 10;20:141. doi: 10.1186/s13054-016-1323-4 (PMC4862118; doi:10.1186/s13054-016-1323-4)
Supplement: Additional file 1: Table E1. — The List of institutional reviewer boards approved the SOS-KANTO 2012 study. Online data supplement. (DOC 42 kb) [file 13054_2016_1323_MOESM1_ESM.doc]

**Nighttime is associated with decreased survival and resuscitation efforts for out-of-hospital cardiac arrests**

Yosuke Matsumura, Taka-aki Nakada, Koichiro Shinozaki, Takashi Tagami, Tomohisa Nomura, Yoshio Tahara, Atsushi Sakurai, Naohiro Yonemoto, Ken Nagao, Arino Yaguchi, Naoto Morimura; SOS-KANTO 2012 study group

***Online data Supplement***

**Table E1. The List of institutional reviewer boards approved the SOS-KANTO 2012 study.**

Tokai University School of Medicine, St. Marianna University School of Medicine, Yokohama Seibu Hospital, Koto Hospital, Saitama Medical Center Advanced Tertiary Medical Center, Kawasaki Municipal Hospital Emergency and Critical Care Center, Yokohama Municipal Citizen’s Hospital, Japanese Red Cross Maebashi Hospital, Juntendo University Urayasu Hospital, Dokkyo Medical University Koshigaya Hospital, Hachioji Medical Center of Tokyo Medical University, Tokyo Women’s Medical University Hospital, Kimitsu Chuo Hospital Chiba University Graduate School of Medicine, Saiseikai Utsunomiya Hospital, Mito Saiseikai General Hospital, Dokkyo Medical University, Yokohama City University Medical Center, National Hospital Organization Yokohama Medical Center, National Disaster Medical Center, Yamanashi Prefectural Central Hospital, Surugadai Nihon University Hospital, Yokohama Rosai Hospital, Showa General Hospital, Nippon Medical School Tamanagayama Hospital, Tokyo Women’s Medical University Yachiyo Medical Center, Awa Regional Medical Center, Toda Chuo General Hospital, Japanese Red Cross Medical Center, St. Luke’s International Hospital Showa University School of Medicine, Totsuka Kyoritsu Hospital, St. Marianna University School of Medicine, National Hospital Organization Mito Medical Center, Tokyo Metropolitan Tama Medical Center, Showa University Fujigaoka Hospital, Gunma University Graduate School of Medicine, Saitama Red Cross Hospital, Tokyo Metropolitan Bokutoh Hospital, Nippon Medical School Hospital, Keio University Hospital, Chiba Emergency Medical Center, Teikyo University School of Medicine, Japanese Red Cross Musashino Hospital, National Center for Global Health and Medicine Hospital, Tokyo Metropolitan Police Hospital, Medical Hospital of Tokyo Medical and Dental University, Juntendo University Nerima Hospital, Nihon University School of Medicine, Toho University Ohashi Medical Center, Saiseikai Yokohamashi Tobu Hospital, Nippon Medical School Musashikosugi Hospital, Tokyo Rosai Hospital, Toho University Omori Medical Center, Hiratsuka City Hospital, Yokosuka Kyosai Hospital, Saiseikai Yokohamashi Nambu Hospital, Nippon Medical School Chiba Hokusoh Hospital, Tokyo Metropolitan Children’s Medical Centre, National Medical Center for Children and Mothers, Chiba Aoba Municipal Hospital, Kuki General Hospital, Matsudo City Hospital, Japanese Red Cross Narita Hospital, Tokyo Bay Urayasu/Ichikawa Medical Center, NTT Medical Center Tokyo, Tokyo Saiseikai Central Hospital, Fuji Heavy Industries Health Insurance Society OTA Memorial Hospital
